# Recent technology advancements in large‐scale DNA assembly

**DOI:** 10.1002/qub2.79

**Published:** 2024-12-17

**Authors:** Jielin Li, Li Cheng, Yingxin Ma, Yizhi Cai, Junbiao Dai

**Affiliations:** ^1^ Shenzhen Key Laboratory of Synthetic Genomics Guangdong Provincial Key Laboratory of Synthetic Genomics Key Laboratory of Quantitative Synthetic Biology Shenzhen Institute of Synthetic Biology Shenzhen Institute of Advanced Technology Chinese Academy of Sciences Shenzhen China; ^2^ University of Chinese Academy of Sciences Beijing China; ^3^ Manchester Institute of Biotechnology University of Manchester Manchester UK; ^4^ Guangdong Laboratory for Lingnan Modern Agriculture Key Laboratory of Synthetic Biology Ministry of Agriculture and Rural Affairs Agricultural Genomics Institute at Shenzhen Chinese Academy of Agricultural Sciences Shenzhen China

The emergence and development of sequencing and DNA synthesis techniques have empowered us with the ability to read and write genomes, thereby enhancing our understanding and manipulation of biological processes. Over the past two decades, numerous genomes ranging from kilobase‐sized viruses to megabase‐sized bacteria and yeast chromosomes have been artificially synthesized [1]. Notably, the Synthetic Yeast Genome Project (Sc2.0) has successfully constructed all the chromosomes of *Saccharomyces cerevisiae*, with a strain containing 7.5 synthetic chromosomes also being generated [2, 3, 4, 5, 6, 7, 8, 9, 10, 11]. It is anticipated that collaborative efforts among scientists worldwide will soon lead to the creation of the first eukaryotic cell harboring a fully synthesized genome. Furthermore, synthetic genomes for higher eukaryotes are also emerging, as exemplified by the ongoing initial phase of the Synthetic Moss Genome Project (SynMoss) [12].

The synthesis of a genome is achieved through a bottom‐up approach, involving the assembly of short oligonucleotides into larger ones. These short oligonucleotides, typically consisting of hundreds of base pairs, are commonly produced using phosphoramidite chemistry or enzymatic synthesis [13]. Subsequently, the oligos are combined together utilizing various in vitro DNA assembly approaches, including restriction endonuclease (RE)‐based and RE‐independent methods, which have been reviewed in detail elsewhere [14, 15, 16]. These in vitro methods can also be employed to assemble large fragments with nearly identical strategies; however, they come with many drawbacks. RE‐based methods, such as BioBrick, Golden Gate, and YeastFab, are suitable for constructing standardized gene element libraries [17, 18, 19]. Despite this suitability, these methods suffer from shortcomings due to the short overhangs produced by REs that result in insufficiently specific binding of numerous fragments and pose challenges for iterative assembly. To address these issues, several RE‐independent methods have been developed, such as the polymerase cycling assembly and Gibson assembly, which have been commonly utilized for concatenating small DNA segments into larger constructs [20, 21]. Nevertheless, they are constrained by labor‐intensive processes and limited fragment length typically within 10–15 kb. In addition, in vitro constructs up to 10 kb may be susceptible to shearing and cause cell incompatibility [22]. Conversely, in vivo assembly offers significant advantages for chromosome‐level assembly. Therefore, for genomes that are frequently on a megabase‐scale level, hierarchical assembly strategies become necessary. This involves starting with in vitro assembly to construct smaller fragments and subsequently assembling them in vivo to generate the entire genome. In this commentary, we summarize the strategies employed in constructing large chromosomes and focus on the current advancements in assembling tens of kilobase‐sized or even megabase‐sized DNA, including in vivo large‐scale assembly strategy and emerging natural DNA‐based chromosome construction (Figure 1). We also discuss the challenges encountered during synthetic genome research along with potential solutions.

**FIGURE 1:**
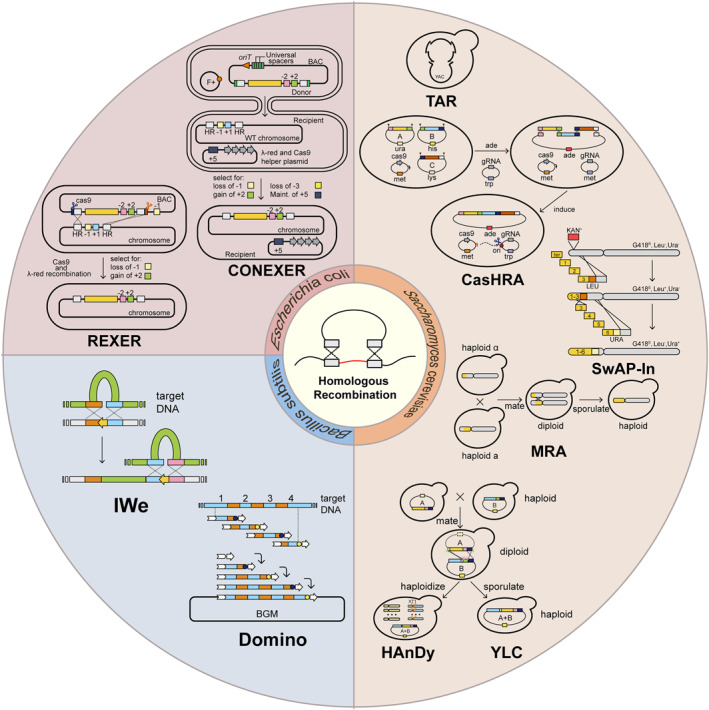
The methods based on homologous recombination in vivo.

*Escherichia coli* (*E. coli*) is widely used as a model organism in various synthetic biology research projects [23]. In the early stages, DNA construction in *E. coli* relied on inefficient RecA‐mediated recombination [24]. The discovery of phage‐encoded recombinant systems, such as the Rac‐encoded RecET system and the Lambda‐encoded red system, has enabled the efficient assembly in *E. coli*. The Rac‐encoded RecET system functions through recombination mediated by RecE and RecT, facilitating effective homologous recombination between two linear DNA molecules [25]. This system has been used for constructing 10 megasynthase secondary metabolite pathways (10–52 kb) of *Photorhabdus luminescens* in *E. coli* [26]. Lambda‐red recombination and its related systems are widely employed in the replacement and assembly of double‐stranded DNA in the *E. coli* genome. The system is mainly composed of the following three genes: λ exo, λ bet, and λ gam, respectively encoding a dsDNA exonuclease Exo, a single‐stranded DNA‐annealing protein Red β and a nuclease inhibitor Gam. The system only requires approximately 50 bp of genome homology and is particularly suitable for short DNA assembly [27].

Owing to the limited effectiveness of strategies mentioned above for the integration of long fragments, a series of derivatization systems have been developed to overcome the barriers of kilobase‐sized and megabase‐sized plasmid construction. Among these, notable systems include REXER (Replicon EXcision Enhanced Recombination) and its iterative repetition GENESIS (GENomE Stepwise Interchange Synthesis), which are used for *E. coli* genome construction [28, 29]. The conjugation coupled with programmed excision for enhanced recombination (CONEXER) and its related assembly methods have also demonstrated significant promise [30]. REXER strictly relies on the CRISPR/Cas9 and Lambda‐red system. Using selection cassette, it has the capability to replace >100 kb *E. coli* genome with synthetic DNA in a single step with high fidelity. Furthermore, it can identify the segments of the synthetic genome that are toxic in *E. coli* [28]. However, careful design of spacers is crucial for REXER as their sequences can greatly impact efficiency. Based on REXER, CONEXER incorporates universal spacers and the origin of transfer, reducing construction time of synthetic chromosomes through combined transfer. CONEXER can be extended to bacterial artificial chromosome (BAC) stepwise insertion synthesis, a megabase‐size DNA fragment scarless iterative assembly approach. This method was successfully employed to assemble the 1.1 Mb human genome in *E. coli*. In addition, this method supports parallelizable and continuous synthesis and enables the construction of a 0.5 Mb *E. coli* genome within approximately 10 days without sequencing verification [30]. These technologies have demonstrated their ability to assemble advanced eukaryotic genomes and will establish the role of *E. coli* as an intermediate platform in synthetic genomics. In addition to rapid and convenient assembly and transfer capabilities, they can also utilize numerous genetic engineering application packages for complete genetic modification of higher animals in *E. coli*, which provides a key foundation for synthetic genomics.

*Bacillus subtilis (B. subtilis)*, known for its high transformability with extracellular DNA, serves as a platform for the synthesis of large DNA molecules. Through its natural competence, single‐stranded extracellular DNA can enter the cytoplasm of *B. subtilis* and be efficiently integrated into the genome via RecA‐mediated homologous recombination. This process enables the cloning and assembly of substantial DNA fragments [31]. Previously demonstrated in 1995, Itaya et al. successfully cloned a 48.5 kb prophage lambda DNA using the *B. subtilis* genome (BGM) as a vector [32]. Subsequently, two construction methods were developed based on BGM: the inchworm elongation (IWe) and the domino. These methods have been employed to facilitate stable incorporation of foreign large‐sized DNA from photosynthetic bacterium with a 3.5 Mb genome, as well as the genomes of mouse mitochondrion and rice chloroplast, respectively [33, 34]. However, these two methods also have many limitations. For example, IWe requires high‐quality continuous DNA templates and is not suitable for de novo chemical synthesis or assembling PCR‐amplified products [33]. In the case of the domino method, there are size restrictions regarding *B. subtilis* transformation for domino DNA; moreover, the activity of endogenous RecA can impact recombination efficiency and specificity [34]. Several conjugated systems are currently being developed to overcome challenges in *B. subtilis*, such as the iREX system, a BGM expression vector containing an inducible RecA and transporting DNA through conjugative transfer [35]. Existing research suggests that *B. subtilis* has shown potential in working with otherwise difficult‐to‐clone DNAs, indicating its suitability as a platform for higher eukaryotic genome assembly [34].

Homologous recombination plays an important role in the assembly of large DNA fragments. *Saccharomyces cerevisiae* is often used to develop platforms for large fragment DNA construction due to its well‐established genetic toolbox and efficient recombination capability. Notably, it can tolerate single chromosomes up to 10 Mb in size, demonstrating its feasibility as a platform for assembling giant fragments [36, 37]. Transformation‐associated recombination (TAR) is a widely used assembly strategy [38, 39], facilitating the cloning of specific chromosome fragments and the construction of yeast artificial chromosomes through the utilization of designed homology arms [40]. This technique enables efficient hierarchical assembly of large fragments following the in vitro assembly technology of short fragments. The remarkable example is the successful construction of the entire *Mycoplasma genitalium* genome from 101 segments by combining the TAR and Gibson assemblies [22]. TAR can also be employed alongside the Golden Gate and meganuclease methods to assemble DNA fragments ranging from 30 to 50 kb [41, 42]. However, during TAR, each round of assembly requires in vitro manipulation of large linear DNA, which may lead to decreased efficiency in stepwise assembly. To address this limitation, a Cas9‐facilitated Homologous Recombination Assembly (CasHRA) has been developed for one‐step assembly of DNA in vivo. The CRISPR system can be used to achieve rapid assembly of the 1.03 Mb minimal genome of *E. coli*, with an efficiency of approximately 65% ± 13%. Moreover, it offers comparable time and effort required for assembling DNAs ranging from hundreds kilobases to one megabase [43].

In the assembly of larger chromosome segments, for example, Sc2.0, the main assembly method is in vivo switching auxotrophies progressively for integration (SwAP‐In). This method relies on the yeast’s homologous recombination ability by alternately utilizing different marker genes to select successfully assembled strains. The synthetic genome was stepwise replaced into *S. cerevisiae* to achieve replacement and assembly of the entire chromosome [44]. In order to better de novo construct arbitrary DNA in yeast, eSwAP‐In (extrachromosomal switching auxotrophies progressively by integration) was developed. Different from SwAP‐In, this technology aims to assemble circular DNA outside the chromosome and has been utilized to construct the 101 kb human genome locus hHPRT1 [45]. While this strategy exhibits high efficiency with fewer rounds of replacement, it can be cumbersome and inefficient once the number of rounds is increased. Therefore, a new strategy named the meiotic recombination‐mediated assembly was developed and first applied to the assembly of *S. cerevisiae* SynXII [46]. This method leverages the unique crossover recombination process during meiosis of yeast cells to combine synthetic chromosome fragments from different strains. Strains containing the combined sequences are then screened for achieving the assembly of large fragments. *Saccharomyces cerevisiae* chromosomes II and V have also applied this strategy, with improvements such as the addition of an I‐SceI site to induce double‐strand breaks and enhance integration efficiency [47, 48]. Recently, He et al. designed a yeast life cycle‐mediated iterative assembly method (termed YLC‐assembly). The assembly mainly relies on the yeast mating–sporulation cycle and the CRISPR/Cas system for iterative editing, eliminating the need for spheroplast fusion or in vitro cumbersome operations such as the purification of large DNA fragments. It has currently achieved in vivo construction of a 95‐kb fragment of yeast essential genes and 1.26 Mb of human immunoglobulin heavy locus DNA. However, due to the sporulation progress, each round of assembly requires a lengthy experimental period (approximately 11 days) [49]. This shortcoming was overcome by the latest method named HAnDy (Haploidization‐based DNA Assembly and Delivery in yeast), which utilizes CRISPR/Cas to program haploidization of diploid strains, greatly improving assembly efficiency [50].

With the efficient strategies mentioned above, yeast can be used to develop low‐cost synthesis methods, such as the emerging tools of natural DNA assembly. Recently, a method called CReATiNG (Cloning, Reprogramming, and Assembling Tiled Natural Genomic DNA) has been reported for constructing synthetic chromosomes using natural components in yeast [51]. Alessandro et al. successfully applied this strategy to rapidly assemble and replace chromosome I with recombined chromosomes derived from different strains and species. Using *S. cerevisiae* as a platform, this work demonstrates a low‐cost and high‐efficiency flexible chromosome assembly method with excellent compatibility that can rectify design flaws through homologous recombination between synthetic and wild‐type chromosomes.

*Saccharomyces cerevisiae* has emerged as a highly promising platform for the synthesis of animal and plant genomes, making significant contributions to initiatives like the international Genome Project (GP)‐Write consortium in 2016 [52]. Notably, the hierarchical assembly approach in yeast was employed to construct mage‐chunks of approximately 100 kb for the recently completed genome assembly of *Physcomitrella patens* semi‐syn18L, which were subsequently transformed into *P. patens* to replace the wildtype sequence [12]. Furthermore, standardization of large fragment assembly techniques in *S. cerevisiae* has been implemented to enhance efficiency in subsequent applications.

The field of synthetic genomics is currently undergoing sustainable development, achieving de novo chromosome synthesis of several single‐celled organisms, with a multitude of emerging strategies for DNA synthesis and assembly [53]. The ability to completely reprogram a chromosome offers profound insights into fundamental topics such as gene functions and regulations, facilitating the introduction of novel designs including genetic codon alteration and chromosomal chimerism. However, most of the existing strategies are applied in bacteria or yeast (Table 1). The synthesis of chromosomes in multicellular organisms poses numerous challenges due to the extensive size of genomes, complex genome structure, and the relatively limited efficiency of homologous recombination. The major limitation is the DNA synthesis, which may involve gigabase‐scale sequences with high guanine‐cytosine content and repeat patterns that pose challenges for accurate assembly and intolerance within donor cells. Traditional chemical methods for polynucleotide synthesis, such as the phosphoramidite oligonucleotide synthesis, have shortages including the inability to generate poly‐repeat sequences and low efficiency in synthesizing fragments beyond ∼200 bp oligonucleotides. In addition, these reactions depend on substantial amount of organic solvents. Relatively, enzymatic DNA synthesis will be a superior potential approach in terms of cost‐effectiveness, accuracy, and synthesis length [13]. On the other hand, building chromosomes from natural DNA is cost‐effective for target sequences that already exist. In addition to the aforementioned method CReATiNG, other potential large‐scale DNA assembly strategies, such as the Cas9‐Assisted Targeting of CHromosome segments (CATCH) and Cas12a‐assisted precise targeted cloning using in vivo Cre‐lox recombination (CAPTURE), also have promising prospects of higher organisms chromosome construction in low cost [55, 56]. Another challenge lies in accurately assembling long stretches of DNA sequences. Customization of recombination fragment end sequences could prevent errors during assembly of scattered short repetitive sequences. The aforementioned strategies for assembling large DNA fragments in vivo provide solutions for synthesizing heterologous genomes in *E. coli* and *S. cerevisiae*. How to deliver chromosome‐sized DNA into recipient cells, particularly for nonmodel organisms, is one of the bottlenecks. While fusing spheroplasts poses risks related to contamination and inefficiency, these concerns could be mitigated by developing a universal host cell capable of accommodating diverse types of DNA molecules. In addition, establishing approaches to achieve assembly of chromosome‐sized DNA in vitro could serve as alternative solutions. Although in vitro assembly has demonstrated the capability to assemble the 144 kb quarter *M. genitalium* genome, numerous obstacles need to be addressed when scaling up to megabase size, including decreased efficiencies associated with increased assembled size and substantial DNA requirements due to lack of DNA amplification steps [22].

**TABLE 1:** Advantages and challenges of aforementioned assembly methods.

Assembly platform	Assembly method	Timing for total constructs	Reported size of largest DNA assembly	Advantages	Challenge and points worth noting
*Escherichia coli*	REXER and GENESIS [28]	4 days for a single integration	∼100 kb each step, 0.5 Mb total	1. Highly efficient and accurate one‐step in vivo genome integration of large synthetic DNA fragment 2. The efficiency is independent of the DNA size to be integrated	1. Specific spacers design is required for each locus 2. Two sequential rounds of competent cell preparation and electroporation are required for each iteration 3. Assembly of potential lethal sequences is difficult 4. The size capacity of each REXER step is limited by the capability to introduce DNA into *E. coli* cells
	CONEXER and BASIS [30]	1 day for a single integration, 10 days for 0.5 Mb continuously	96 kb each steps, 1.1 Mb total	1. Universal spacers enable scarless replacement 2. Enable iterative recombination rapidly, scalably and in parallel 3. Minimize crossovers between the synthetic DNA and the genome 4. Identify problematic synthetic sequences rapidly and precisely	1. Synthetic DNA should be designed carefully and assembly of potential lethal sequences is difficult 2. The design of viable genomes and the fixing of disallowed synthetic sequences continue to pose challenges
*Bacillus subtilis*	IWe [33]	NA	3.5 Mb	Compatibility with megacloning	Long continuous DNA (>100 kb) with high quality and high concentration is required
	Domino clones [34]	NA	134.5 kb	1. The clone sizes can vary depending on the design and preparation method, as long as it meets the requirements for dominoes 2. The suitable BGM vector can also be used with other conventional vectors	1. Restrictions on the size of *B. subtilis* transformation 2. The activity of endogenous RecA can impact recombination efficiency and specificity 3. Certain levels of deterioration of the GC‐rich guest DNA appeared if the size exceeded several hundred kbp [54]
*Saccharomyces cerevisiae*	TAR [22]	NA	1.08 Mb	Assembly process only requires in vivo homologous recombination in yeast	1. Requires in vitro manipulation of large linear DNA each round 2. Efficient import and export steps of large DNA to host cells are required 3. Low efficiency of DNA larger than 100 kb in size
	CasHRA [43]	9 days for entire process	1.03 Mb	1. Large circular DNAs (>100 kb) can be directly used in a one‐step DNA assembly process in vivo 2. The time and effort required to assemble DNAs with different sizes were similar 3. The assembly efficiency is high (60%–80%) 4. The efficient elimination of the guide RNA expression vector enables iterative assembly	1. Length and sequences of assembly overlaps must be carefully considered to ensure the efficiency 2. Yeast spheroplasts fusion is inefficient
	SwAP‐In [44]	NA	0.24–0.77 Mb	1. Avoid the direct assembly of large DNA 2. Enable step‐by‐step testing of the functions of the newly integrated sequences	1. Stepwise assembly is time‐consuming 2. Less efficient once the number of rounds is extended 3. Special sites are required for insertion and the segmentation should be considered
	MRA [46]	NA	1.1 Mb	1. Improve SwAP‐In efficiency and is suitable for large fragments integration 2. Synthetic fitness defects between different synthetic regions can be identified	Meiotic recombination is relatively inefficient
	YLC [49]	∼11 days each round	1.26 Mb	1. The workflow is easy to operate 2. The assembly accuracy is high 3. Avoid the transformation of large DNA fragments into yeast and the manipulation of DNA molecules in vitro	The process of mating and sporulation is relatively time‐consuming
	HAnDy [50]	∼6 days each round	1.024 Mb	1. Haploidization helps to bypass the process of meiosis 2. Enable convenient iterative assembly 3. Enable selected delivery of the megabase DNA to different unmodified recipient strains	CRISPR/Cas9‐mediated haploidization could only be achieved in the specific engineered strain

Abbreviations: BASIS, bacterial artificial chromosome (BAC) stepwise insertion synthesis; BGM, *B. subtilis* genome; CasHRA, Cas9‐facilitated Homologous Recombination Assembly; CONEXER, conjugation coupled with programmed excision for enhanced recombination; GC, guanine‐cytosine; GENESIS, GENomE Stepwise Interchange Synthesis; HAnDy, Haploidization‐based DNA Assembly and Delivery in yeast; IWe, inchworm elongation; MRA, meiotic recombination‐mediated assembly; NA, not applicable; REXER, Replicon EXcision Enhanced Recombination; SwAP‐In, switching auxotrophies progressively for integration; TAR, transformation‐associated recombination; YLC, yeast life cycle.

The advancement of DNA assembly techniques offers promising opportunities for various fields of research and applications. By introducing tens to thousands of edits into mammalian genome through de novo synthesis, it facilitates to reveal the functions of genetic elements and opens up new possibilities for building human disease models in research [57]. In conjunction with the heterologous expression, it becomes feasible to assemble and integrate biosynthetic gene clusters that yield valuable compounds or materials into target cells [58]. Similarly, improved crops with increased yield or resistance to diseases and pests would be achieved by integration of genetic resources [59]. Moreover, with the progress in technological development, the cost of constructing and testing chromosome‐sized DNA is expected to undergo a significant decrease, which makes large‐scale genomic projects more feasible and accessible to a wider range of researchers.

## AUTHOR CONTRIBUTIONS

**Jielin Li**: Writing—original draft; writing—review & editing. **Li Cheng**: Writing—original draft; writing—review & editing. **Yingxin Ma**: Writing—review & editing. **Yizhi Cai**: Writing—review & editing. **Junbiao Dai**: Project administration; supervision; writing—review & editing.

## CONFLICT OF INTEREST STATEMENT

The authors declare no conflicts of interest.

## ETHICS STATEMENT

This manuscript does not involve a research protocol requiring approval by the relevant institutional review board or ethics committee.

## Data Availability

The authors confirm that the data supporting the findings of this study are available within the article.

## References

[1] Venter JC , Glass JI , Hutchison CA , Vashee S . Synthetic chromosomes, genomes, viruses, and cells. Cell. 2022;185(15):2708–2724. 35868275 10.1016/j.cell.2022.06.046 PMC9347161

[2] Blount BA , Lu X , Driessen MRM , Jovicevic D , Sanchez MI , Ciurkot K , et al. Synthetic yeast chromosome *XI* design provides a testbed for the study of extrachromosomal circular DNA dynamics. Cell Genom. 2023;3(11):100418. 38020971 10.1016/j.xgen.2023.100418 PMC10667340

[3] Foo JL , Kitano S , Susanto AV , Jin Z , Lin Y , Luo Z , et al. Establishing chromosomal design‐build‐test‐learn through a synthetic chromosome and its combinatorial reconfiguration. Cell Genom. 2023;3(11):100435. 38020970 10.1016/j.xgen.2023.100435 PMC10667554

[4] Lauer S , Luo J , Lazar‐Stefanita L , Zhang W , McCulloch LH , Fanfani V , et al. Context‐dependent neocentromere activity in synthetic yeast chromosome *VIII* . Cell Genom. 2023;3(11):100437. 38020969 10.1016/j.xgen.2023.100437 PMC10667555

[5] Luo J , Vale‐Silva LA , Raghavan AR , Mercy G , Heldrich J , Sun X , et al. Synthetic chromosome fusion: effects on mitotic and meiotic genome structure and function. Cell Genom. 2023;3(11):100439. 38020967 10.1016/j.xgen.2023.100439 PMC10667551

[6] McCulloch LH , Sambasivam V , Hughes AL , Annaluru N , Ramalingam S , Fanfani V , et al. Consequences of a telomerase‐related fitness defect and chromosome substitution technology in yeast *synIX* strains. Cell Genom. 2023;3(11):100419. 38020974 10.1016/j.xgen.2023.100419 PMC10667316

[7] Schindler D , Walker RSK , Jiang S , Brooks AN , Wang Y , Müller CA , et al. Design, construction, and functional characterization of a tRNA neochromosome in yeast. Cell. 2023;186(24):5237–5253.e22. 37944512 10.1016/j.cell.2023.10.015

[8] Shen Y , Gao F , Wang Y , Wang Y , Zheng J , Gong J , et al. Dissecting aneuploidy phenotypes by constructing Sc2.0 chromosome *VII* and SCRaMbLEing synthetic disomic yeast. Cell Genom. 2023;3(11):100364. 38020968 10.1016/j.xgen.2023.100364 PMC10667312

[9] Williams TC , Kroukamp H , Xu X , Wightman ELI , Llorente B , Borneman AR , et al. Parallel laboratory evolution and rational debugging reveal genomic plasticity to *S. cerevisiae* synthetic chromosome *XIV* defects. Cell Genom. 2023;3(11):100379. 38020977 10.1016/j.xgen.2023.100379 PMC10667330

[10] Zhang W , Lazar‐Stefanita L , Yamashita H , Shen MJ , Mitchell LA , Kurasawa H , et al. Manipulating the 3D organization of the largest synthetic yeast chromosome. Mol Cell. 2023;83(23):4424–4437.e5. 37944526 10.1016/j.molcel.2023.10.015

[11] Zhao Y , Coelho C , Hughes AL , Lazar‐Stefanita L , Yang S , Brooks AN , et al. Debugging and consolidating multiple synthetic chromosomes reveals combinatorial genetic interactions. Cell. 2023;186(24):5220–5236.e16. 37944511 10.1016/j.cell.2023.09.025

[12] Chen LG , Lan T , Zhang S , Zhao M , Luo G , Gao Y , et al. A designer synthetic chromosome fragment functions in moss. Nat Plants. 2024;26(2):1–12. 10.1038/s41477-023-01595-7 38278952

[13] Hoose A , Vellacott R , Storch M , Freemont PS , Ryadnov MG . DNA synthesis technologies to close the gene writing gap. Nat Rev Chem. 2023;7(3):144–161. 36714378 10.1038/s41570-022-00456-9 PMC9869848

[14] Casini A , Storch M , Baldwin GS , Ellis T . Bricks and blueprints: methods and standards for DNA assembly. Nat Rev Mol Cell Biol. 2015;16(9):568–576. 26081612 10.1038/nrm4014

[15] Chao R , Yuan Y , Zhao H . Recent advances in DNA assembly technologies. FEMS Yeast Res. 2015;15(1):1–9. 10.1111/1567-1364.12171 PMC4257898 24903193

[16] Ellis T , Adie T , Baldwin GS . DNA assembly for synthetic biology: from parts to pathways and beyond. Integr Biol. 2011;3(2):109–118. 10.1039/c0ib00070a 21246151

[17] Shetty RP , Endy D , Knight TF . Engineering BioBrick vectors from BioBrick parts. J Biol Eng. 2008;2(1):5. 18410688 10.1186/1754-1611-2-5 PMC2373286

[18] Agmon N , Mitchell LA , Cai Y , Ikushima S , Chuang J , Zheng A , et al. Yeast Golden Gate (yGG) for the efficient assembly of *S. cerevisiae* transcription units. ACS Synth Biol. 2015;4(7):853–859. 25756291 10.1021/sb500372z

[19] Guo Y , Dong J , Zhou T , Auxillos J , Li T , Zhang W , et al. YeastFab: the design and construction of standard biological parts for metabolic engineering in *Saccharomyces cerevisiae* . Nucleic Acids Res. 2015;43(13):e88. 25956650 10.1093/nar/gkv464 PMC4513847

[20] Gibson DG , Young L , Chuang RY , Venter JC , Hutchison CA , Smith HO . Enzymatic assembly of DNA molecules up to several hundred kilobases. Nat Methods. 2009;6(5):343–345. 19363495 10.1038/nmeth.1318

[21] Stemmer WPC , Crameri A , Ha KD , Brennan TM , Heyneker HL . Single‐step assembly of a gene and entire plasmid from large numbers of oligodeoxyribonucleotides. Gene. 1995;164(1):49–53. 7590320 10.1016/0378-1119(95)00511-4

[22] Gibson DG , Benders GA , Andrews‐Pfannkoch C , Denisova EA , Baden‐Tillson H , Zaveri J , et al. Complete chemical synthesis, assembly, and cloning of a *Mycoplasma genitalium* genome. Science. 2008;319(5867):1215–1220. 18218864 10.1126/science.1151721

[23] Adams BL . The next generation of synthetic biology chassis: moving synthetic biology from the laboratory to the field. ACS Synth Biol. 2016;5(12):1328–1330. 27665861 10.1021/acssynbio.6b00256

[24] O’Connor M , Peifer M , Bender W . Construction of large DNA segments in *Escherichia coli* . Science. 1989;244(4910):1307–1312. 2660262 10.1126/science.2660262

[25] Zhang Y , Buchholz F , Muyrers JPP , Stewart AF . A new logic for DNA engineering using recombination in *Escherichia coli* . Nat Genet. 1998;20(2):123–128. 9771703 10.1038/2417

[26] Fu J , Bian X , Hu S , Wang H , Huang F , Seibert PM , et al. Full‐length RecE enhances linear‐linear homologous recombination and facilitates direct cloning for bioprospecting. Nat Biotechnol. 2012;30(5):440–446. 22544021 10.1038/nbt.2183

[27] Wannier TM , Ciaccia PN , Ellington AD , Filsinger GT , Isaacs FJ , Javanmardi K , et al. Recombineering and MAGE. Nat Rev Methods Primers. 2021;1(1):1–24. 10.1038/s43586-020-00006-x PMC9083505 35540496

[28] Robertson WE , Funke LFH , de la Torre D , Fredens J , Wang K , Chin JW . Creating custom synthetic genomes in *Escherichia coli* with REXER and GENESIS. Nat Protoc. 2021;16(5):2345–2380. 33903757 10.1038/s41596-020-00464-3 PMC11585970

[29] Fredens J , Wang K , de la Torre D , Funke LFH , Robertson WE , Christova Y , et al. Total synthesis of *Escherichia coli* with a recoded genome. Nature. 2019;569(7757):514–518. 31092918 10.1038/s41586-019-1192-5 PMC7039709

[30] Zürcher JF , Kleefeldt AA , Funke LFH , Birnbaum J , Fredens J , Grazioli S , et al. Continuous synthesis of *E. coli* genome sections and Mb‐scale human DNA assembly. Nature. 2023;619(7970):555–562. 37380776 10.1038/s41586-023-06268-1 PMC7614783

[31] Juhas M , Ajioka JW . Integrative bacterial artificial chromosomes for DNA integration into the *Bacillus subtilis* chromosome. J Microbiol Methods. 2016;125:1–7. 27033694 10.1016/j.mimet.2016.03.017

[32] Itaya M . Toward a bacterial genome technology: integration of the *Escherichia coli* prophage lambda genome into the *Bacillus subtilis* 168 chromosome. Mol Gen Genet. 1995;248(1):9–16. 7651332 10.1007/BF02456608

[33] Itaya M , Tsuge K , Koizumi M , Fujita K . Combining two genomes in one cell: stable cloning of the Synechocystis PCC6803 genome in the *Bacillus subtilis* 168 genome. Proc Natl Acad Sci USA. 2005;102(44):15971–15976. 16236728 10.1073/pnas.0503868102 PMC1276048

[34] Itaya M , Fujita K , Kuroki A , Tsuge K . Bottom‐up genome assembly using the *Bacillus subtilis* genome vector. Nat Methods. 2008;5(1):41–43. 18066072 10.1038/nmeth1143

[35] Ogawa T , Iwata T , Kaneko S , Itaya M , Hirota J . An inducible recA expression *Bacillus subtilis* genome vector for stable manipulation of large DNA fragments. BMC Genom. 2015;16(1):209. 10.1186/s12864-015-1425-4 PMC4374399 25879542

[36] Shao Y , Lu N , Cai C , Zhou F , Wang S , Zhao Z , et al. A single circular chromosome yeast. Cell Res. 2019;29(1):87–89. 30559437 10.1038/s41422-018-0110-y PMC6318310

[37] Gibson DG . Gene and genome construction in yeast. Curr Protoc Mol Biol. 2011;94(1):3.22.1–3.22.17. 10.1002/0471142727.mb0322s94 21472698

[38] Kouprina N , Larionov V . TAR cloning: insights into gene function, long‐range haplotypes and genome structure and evolution. Nat Rev Genet. 2006;7(10):805–812. 16983376 10.1038/nrg1943

[39] Larionov V , Kouprina N , Solomon G , Barrett JC , Resnick MA . Direct isolation of human *BRCA2* gene by transformation‐associated recombination in yeast. Proc Natl Acad Sci USA. 1997;94(14):7384–7387. 9207100 10.1073/pnas.94.14.7384 PMC23830

[40] Jiang S , Tang Y , Xiang L , Zhu X , Cai Z , Li L , et al. Efficient *de novo* assembly and modification of large DNA fragments. Sci China Life Sci. 2022;65(7):1445–1455. 34939159 10.1007/s11427-021-2029-0

[41] Mitchell LA , Chuang J , Agmon N , Khunsriraksakul C , Phillips NA , Cai Y , et al. Versatile genetic assembly system (VEGAS) to assemble pathways for expression in *S. cerevisiae* . Nucleic Acids Res. 2015;43(13):6620–6630. 25956652 10.1093/nar/gkv466 PMC4513848

[42] Kuijpers NG , Chroumpi S , Vos T , Solis‐Escalante D , Bosman L , Pronk JT , et al. One‐step assembly and targeted integration of multigene constructs assisted by the *I‐SceI* meganuclease in *Saccharomyces cerevisiae* . FEMS Yeast Res. 2013;13(8):769–781. 24028550 10.1111/1567-1364.12087 PMC4068284

[43] Zhou J , Wu R , Xue X , Qin Z . CasHRA (Cas9‐facilitated homologous recombination assembly) method of constructing megabase‐sized DNA. Nucleic Acids Res. 2016;44(14):e124. 27220470 10.1093/nar/gkw475 PMC5001600

[44] Richardson SM , Mitchell LA , Stracquadanio G , Yang K , Dymond JS , DiCarlo JE , et al. Design of a synthetic yeast genome. Science. 2017;355(6329):1040–1044. 28280199 10.1126/science.aaf4557

[45] Mitchell LA , McCulloch LH , Pinglay S , Berger H , Bosco N , Brosh R , et al. *De novo* assembly and delivery to mouse cells of a 101 kb functional human gene. Genetics. 2021;218(1):iyab038. 33742653 10.1093/genetics/iyab038 PMC8128383

[46] Zhang W , Zhao G , Luo Z , Lin Y , Wang L , Guo Y , et al. Engineering the ribosomal DNA in a megabase synthetic chromosome. Science. 2017;355(6329):eaaf3981. 28280149 10.1126/science.aaf3981

[47] Shen Y , Wang Y , Chen T , Gao F , Gong J , Abramczyk D , et al. Deep functional analysis of *synII*, a 770‐kilobase synthetic yeast chromosome. Science. 2017;355(6329):eaaf4791. 28280153 10.1126/science.aaf4791 PMC5390853

[48] Xie ZX , Li BZ , Mitchell LA , Wu Y , Qi X , Jin Z , et al. “Perfect” designer chromosome *V* and behavior of a ring derivative. Science. 2017;355(6329):eaaf4704. 28280151 10.1126/science.aaf4704

[49] He B , Ma Y , Tian F , Zhao GR , Wu Y , Yuan YJ . YLC‐assembly: large DNA assembly via yeast life cycle. Nucleic Acids Res. 2023;51(15):8283–8292. 37486765 10.1093/nar/gkad599 PMC10450165

[50] Ma Y , Su S , Fu Z , Zhou C , Qiao B , Wu Y , et al. Convenient synthesis and delivery of a megabase‐scale designer accessory chromosome empower biosynthetic capacity. Cell Res. 2024;8(4):1–14. 10.1038/s41422-024-00934-3 PMC10978979 38332200

[51] Coradini ALV , Ville CN , Krieger ZA , Roemer J , Hull C , Yang S , et al. Building synthetic chromosomes from natural DNA. Nat Commun. 2023;14(1):8337. 38123566 10.1038/s41467-023-44112-2 PMC10733283

[52] Boeke JD , Church G , Hessel A , Kelley NJ , Arkin A , Cai Y , et al. The genome project‐write. Science. 2016;353(6295):126–127. 27256881 10.1126/science.aaf6850

[53] Zhang XE , Liu C , Dai J , Yuan Y , Gao C , Feng Y , et al. Enabling technology and core theory of synthetic biology. Sci China Life Sci. 2023;6(8):1–44. 10.1007/s11427-022-2214-2 PMC9907219 36753021

[54] Ohtani N , Hasegawa M , Sato M , Tomita M , Kaneko S , Itaya M . Serial assembly of *Thermus* megaplasmid DNA in the genome of *Bacillus subtilis* 168: a BAC‐based domino method applied to DNA with a high GC content. Biotechnol J. 2012;7(7):867–876. 22553167 10.1002/biot.201100396

[55] Jiang W , Zhao X , Gabrieli T , Lou C , Ebenstein Y , Zhu TF . Cas9‐Assisted Targeting of CHromosome segments CATCH enables one‐step targeted cloning of large gene clusters. Nat Commun. 2015;6(1):8101. 26323354 10.1038/ncomms9101 PMC4569707

[56] Enghiad B , Huang C , Guo F , Jiang G , Wang B , Tabatabaei SK , et al. Cas12a‐assisted precise targeted cloning using *in vivo* Cre‐lox recombination. Nat Commun. 2021;12(1):1171. 33608525 10.1038/s41467-021-21275-4 PMC7896053

[57] Zhang W , Golynker I , Brosh R , Fajardo A , Zhu Y , Wudzinska AM , et al. Mouse genome rewriting and tailoring of three important disease loci. Nature. 2023;623(7986):423–431. 37914927 10.1038/s41586-023-06675-4 PMC10632133

[58] Li R , Li Z , Ma K , Wang G , Li W , Liu H , et al. Strategy for efficient cloning of biosynthetic gene clusters from fungi. Sci China Life Sci. 2019;62:1087–1095. 31209796 10.1007/s11427-018-9511-7

[59] Bailey‐Serres J , Parker JE , Ainsworth EA , Oldroyd GED , Schroeder JI . Genetic strategies for improving crop yields. Nature. 2019;575(7781):109–118. 31695205 10.1038/s41586-019-1679-0 PMC7024682

